# A Network Pharmacological Approach to Reveal the Pharmacological Targets and Its Associated Biological Mechanisms of Prunetin-5-O-Glucoside against Gastric Cancer

**DOI:** 10.3390/cancers13081918

**Published:** 2021-04-15

**Authors:** Preethi Vetrivel, Rajeswari Murugesan, Pritam Bhagwan Bhosale, Sang Eun Ha, Hun Hwan Kim, Jeong Doo Heo, Gon Sup Kim

**Affiliations:** 1 Research Institute of Life Science and College of Veterinary Medicine, Gyeongsang National University, Gazwa, Jinju 52828, Korea; preethivetrivel05@gmail.com (P.V.); shelake.pritam@gmail.com (P.B.B.); sangdis2@naver.com (S.E.H.); shark159753@naver.com (H.H.K.); 2 Department of Biochemistry, Biotechnology and Bioinformatics, Avinashilingam Institute for Home Science and Higher Education for Women, Coimbatore 641043, India; rajeshwari_bc@avinuty.ac.in; 3 Gyeongnam Department of Environment Toxicology and Chemistry, Toxicity Screening Research Center, Korea Institute of Toxicology, Munsan-eup, Gyeongnam, Jinju 52834, Korea; jdher@kitox.re.kr

**Keywords:** gastric cancer, network pharmacology, biomarkers, flavonoids, molecular binding, pathway analysis

## Abstract

**Simple Summary:**

Identification of pharmacological targets in cancer provides a major walkthrough toward treatment strategies. The present research adopted a network pharmacology approach utilizing a flavonoid glucoside prunetin-5-O-glucoside (PG) compound against gastric cancer. The correlative targets were analyzed using Swiss target prediction and DiGeNET databases. Functional enrichment and significant pathways enriched were predicted for the targets to associate its biological mechanisms with cancer. Protein interaction network and cluster analysis was performed using Search Tool for the Retrieval of Interacting Genes/Proteins (STRING). Our analysis revealed three core targets among the clustered modules that plays a crucial role in relation with cancer. With this information, the core targets were examined for the binding affinity with PG using molecular docking analysis and validations on the protein targets was performed using western blot analysis and Human Protein Atlas. Our analysis through comprehensive network pharmacology resulted in the prediction of three core targets of PG that can be significant biomarkers against gastric cancer.

**Abstract:**

Gastric cancer (GC) is an aggressive malignancy with increased mortality rate and low treatment options. Increasing evidence suggests that network pharmacology will be a novel method for identifying the systemic mechanism of therapeutic compounds in diseases like cancer. The current study aimed to use a network pharmacology approach to establish the predictive targets of prunetin-5-O-glucoside (PG) against gastric cancer and elucidate its biological mechanisms. Primarily, genes associated with the pathogenesis of GC was identified from the DiGeNET database and targets of PG was obtained from the Swiss target prediction database. In total, 65 correlative hits were identified as anti-gastric cancer targets of PG. Functional enrichment and pathway analysis revealed significant biological mechanisms of the targets. Interaction of protein network and cluster analysis using STRING resulted in three crucial interacting hub targets namely, HSP90AA1, CDK2, and MMP1. Additionally, the in vitro cytotoxic potential of PG was assessed on three gastric cancer cells (AGS, MKN-28, and SNU-484). Furthermore, the crucial targets were validated using molecular docking, followed by their expressions being evaluated by western blot and Human Protein Atlas. The findings indicate that the pharmacological action of PG against GC might be associated with the regulation of three core targets: HSP90AA1, CDK2, and MMP1. Thus, the network pharmacology undertaken in the current study established the core active targets of PG, which may be extensively applied with further validations for treatment in GC.

## 1. Introduction

Gastric cancer (GC) is one among common malignant tumors affecting the stomach and digestive system. It remains a prevalent disease globally with a very poor prognosis rate and declining successful treatment options up until now [1]. The survival rate of cancer prognosis is dismal, with less than 20% due to factors such as late diagnosis [2]. With the primary cause of incidence being *Helicobacter pylori* infection, other casual factors may also contribute to the malignancy such as poor diet and obesity [3]. Though the exact cause of GC remains unclear, the pathogenesis is similar to that of other malignant tumors with a multi-step comprehensive disease [4]. The understanding of molecular pathways and alterations in signaling mechanisms of the tumor cells through inhibitors may lead to better improvement in the prognosis [5].

As a heterogeneous disease, significant treatment preferences for cancer require a distinct understanding of its mechanism [6]. In the conditions of the cancerous growth of cells from healthy cells, usually multiple genes and their products participate in the transformation [7]. However, focusing multiple targets or multipathway treatment is relatively difficult to pursue the exact mechanism of action of a drug in conventional experiments [8]. The new generation of drugs focus on targeting specific proteins that are expressed based on cancer type [9]. Understanding the targets and their mechanism of action can be considered remarkably effective as it gives a clear insight for treatment. Additionally, target specific therapy also minimizes the side effect of conventional cytotoxic drugs [10]. The novel and innovative method of understanding the disease state and its targets prior provides a roadmap that directs toward drug development by targeted therapy [11].

Network pharmacology is a method of predicting targets against a particular disease through the available biomedical data in system biology and poly-pharmacology [12]. It is a multidisciplinary approach that integrates computational biology, network analysis, and uses multitarget research strategies [13]. It employs a classical approach in bioinformatics that aids in discovering the underlying mechanisms between the drug compounds and their putative targets [14]. In targeted therapy, network pharmacology can describe the complex relationships among the biological system through network component analysis and determines the synergistic effects in cancer treatment [15].

Intriguingly, naturally sourced active ingredients have been found with original pharmacological activities that can achieve potent efficacy as a medication in treating disorders [16]. Introducing these bioactive components as a novel reliable therapeutic element against different types of human cancer can be effective based on their selective molecular targets [17]. Well-known derivatives of plant source that have been reported with active therapeutic properties against many malignancies are the ‘flavonoids’ [18]. Most of the flavonoids exist in the glycoside form as a dietary nutrient intake and undergoes deglycosylation to aglycone upon consumption [19]. Epidemiological studies on the biological activity of flavonoids has shown proven benefits of protection against cancer development through various mechanisms [20]. Prunetin 5-O-glucoside (PG), also termed as prunetionoside, is a glycosylated flavonoid that has been characterized to date in few sources, namely *Prunus ceruses* and *Betula schmidtii* [21,22]. However, there has been no extensive study on the individual biological mechanism of the compound to date.

**Figure 1:**
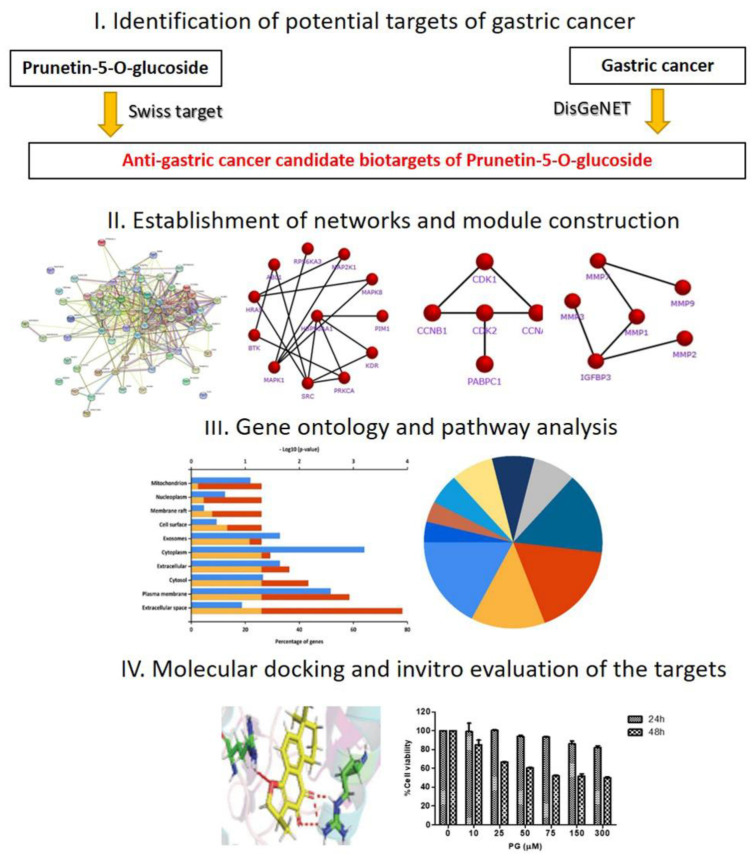
Schematic flowchart designed for the current study to investigate the bio targets and molecular mechanism of prunetin-5-O-glucoside to treat gastric cancer through a network pharmacology approach.

Therefore, the current study aimed to use a network pharmacology approach to establish the pharmacological active targets of prunetin 5-O-glucoside against gastric cancer. In the investigation, a comprehensive network pharmacology strategy and molecular docking approach was used in combination with preliminary in vitro validations to examine the molecular mechanisms. The study is framed as a three-phase workflow stated below: (i) Identification of the potential targets of PG based on its association with GC through retrieval from databases; (ii) Investigation of the key role of the identified targets through functional enrichment and pathway analysis by Gene Ontology (GO) terms; (iii) Establishment of the core targets based on the interaction relationships by network analysis; and (iv) Validation of the potential targets by molecular docking verification and in vitro assessment. A schematic diagram of the integrated process is displayed in Figure 1.

## 2. Materials and Methods

### 2.1. Data Collection and Preparation

The screening of targets against gastric cancer (GC) hits and the relative candidate targets of prunetin-5-O-glucoside (PG) were identified based on databases. The current study used target prediction database Swiss Target Prediction (www.swisstargetprediction.ch, accessed on 30 September 2020) to predict the putative targets of PG. Following this, the target hits associated with GC were derived from the DisGeNET database (http://www.disgenet.org/, accessed on 2 October 2020) using the keyword search “gastric cancer”. The overlapped GC hits with that of the PG targets were considered as anti-gastric cancer PG targets and considered for further analysis.

### 2.2. Gene Ontology (GO) Analysis and Pathway Enrichment on the Identifiable Targets

Gene Ontology and functional pathway enrichment of the obtained targets were performed by FunRich software v.3.1.4 (http://www.funrich.org, accessed on 6 October 2020) using the Reactome database for annotations. GO terms and pathways with false discovery rate (FDR) < 0.01 were picked and defined as the enriched terms and pathways. Each enriched GO term was plotted as a graph in groups of three categories: cellular component, biological process, and molecular function. The top ten significant pathways enriched were chosen based on a Reactome pathway analysis and a doughnut chart was constructed using the FunRich software.

### 2.3. Establishment of STRING Network and Module Construction of Gastric Cancer Targets

Protein-protein interaction (PPI) data of the obtained 65 targets were extracted from the Search Tool for the Retrieval of Interacting Genes/Proteins (STRING, https://string-db.org/, accessed on 12 October 2020). The STRING database provides the interaction of different protein to protein co-relation and its interactive level based on confidence scoring. The identified 65 anti-gastric cancer targets were all inputted into the STRING interaction database with the selection of Homo sapiens category for visualization of an interactive network. The confidence score for the construction of an interactive network was set up with a medium score of 0.4 to 0.7, respectively. Furthermore, the PPI network was subjected to a K-means algorithm and the distinct clustered networks were identified. The clustered networks were input into FunRich software for module construction.

### 2.4. Molecular Docking Analysis

For the evaluation of the putative targets predicted from the network analysis, molecular docking analysis was performed using Glide of Schrodinger-Maestro v.8.5. Primarily, the three-dimensional (3D) structure of PG was obtained from PubChem (pubchem.ncbi.nlm.nih.gov, accessed on 10 November 2020) and minimized. Additionally, the 3D structure of the potential targets was downloaded from the PDB database (www.rcsb.org, accessed on 10 November 2020). All protein structures were processed with receptor grids in order to get various binding poses for the ligand on its active sites. The best ligand fit pose was obtained based on the least Glide score and binding energy was calculated by Schrodinger Prime using molecular mechanics generalized born surface area (MM-GBSA). Furthermore, the amino acid interaction within the protein targets and the ligand were visualized in LigPlot using Schrodinger-Maestro v.8.5, New York, NY, USA.

### 2.5. Pharmacological Study by In Vitro Cytotoxicity Assessment

The human gastric cancer cell lines AGS, MKN-28, SNU-484, and human keratinocyte HaCaT cells were obtained from the Korea cell line bank (Seoul, Korea). After arrival, the gastric cancer cells were cultured in Roswell Park Memorial Institute (RPMI)1640 medium and HaCaT cells in Dulbecco’s Modified Eagle Medium (DMEM) medium supplemented with 10% heat inactivated fetal bovine serum (FBS); 100 U/mL penicillin and 100 μg/mL streptomycin was used as antibiotics. The cells were maintained at 37 °C in a humidified atmosphere of 95% air and 5% CO_2_. The compound prunetinoside with ≥95% purity was purchased from EnsolBio sciences (Daejeon, Korea). AGS, MKN-28, and HaCaT Cells were grown on a 96-well plate seeded at a density of 1 × 10^4^, whereas SNU-484 were seeded at 2 × 10^4^. The grown cells were treated with different doses 0, 10, 25, 50, 75, 150, and 300 µM of PG and incubated at two different time intervals of 24 h and 48 h. Subsequently, the cell viability was performed using 3-(4,5-dimethylthiazol-2-yl)-2,5-diphenyl tetrazolium bromide (MTT) assay upon incubation for 3 h in the dark. The formation of formazan crystals was measured after solubilization in DMSO at 540 nm by spectrophotometry. The amount of the viable cell percentage was calculated in comparison with that of the untreated cells.

### 2.6. Observation of Morphological Changes and DAPI Fluorescent Staining

AGS cells were seeded at a density of 4 × 10^5^ cells per well in a 6-well culture plates and allowed to grow up to 70% confluency. Following this, cells were treated with distinct concentrations of PG (0, 50, 75, and 150 µM) and incubated for 48 h. After 48 h of incubation, the treated cells were subjected to 1X PBS washing, and then followed by fixing using 37% formaldehyde for at least 15 min in room temperature. The fixed cells were further washed with PBS and stained by 4′, 6-diamidino-2-phenylindole (DAPI; Vectashield H-1500; Vector Laboratories, Burlingame, CA, USA). The nuclear morphology of the stained cells were viewed at 780–800 nm under an Olympus FV1000 MPE microscope (Tokyo, Japan). The images were visualized using the software Olympus Fluoview viewer (ver.4.2b, Olympus Global, Tokyo, Japan).

### 2.7. Validation of the Target Gene Expression Levels in Gastric Cancer Using GEPIA

The expression levels of the identified target genes were analyzed through the GEPIA website (gene expression profiling interactive analysis, http://gepia.cancerpku.cn/index.html, accessed on 25 March 2021). GEPIA is a web-based platform with tremendous thousands of expression profiles that allows for interactive expression analysis, patient survival analysis, and gene detection, which aids in disease prognosis and therapeutic discovery process [23]. The mRNA expression levels of the three targets (CDK2, MMP1, and HSP90AA1) were searched under the dataset selection STAD (stomach adenocarcinoma). The total number of sample comparison obtained for the expression levels were 408 STAD samples and 211 non-tumor normal stomach samples. The criteria for obtaining the expression of the mRNA were subjected to Log_2_FC less than 2 and a *p*-value of 0.01 was considered as the significant range. The expression level of the target genes between normal and tumor samples were viewed by constructing a boxplot.

### 2.8. Validation of Target Protein Expression Using Western Blot Assay

To identify the in vitro protein expression of the crucial targets, western blot was performed. AGS gastric cancer cells were seeded at a density of 5 × 10^5^ per well on a 60 mm plate and treated with PG at indicated concentrations (0, 50, and 75) for 48 h at 37 °C. The cells were harvested and the total protein from each group was extracted using radioimmuno-precipitation assay (RIPA) buffer (iNtRON Biotechnology, Seoul, Korea) containing protease and phosphatase inhibitors. The concentration of protein present in each group was determined using the Pierce™ BCA assay (Thermo Fisher Scientific, Rockford, IL, USA). Protein samples were separated by 8–15% sodium dodecyl sulfate polyacrylamide gel electrophoresis (SDS-PAGE) based on its molecular weight, followed by transfer on to a polyvinylidene fluoride membrane (PVDF). The membrane was further blocked in 5% bovine serum albumin (BSA) solution for about 1 h at room temperature. Upon blocking, each protein of interest was incubated with their respective primary antibodies: CDK2 (1:1000), MMP1 (1:1000), HSP90 (1:1000), and β-Actin (1:1000) at 4 °C overnight. Incubated membranes were washed with TBS-T buffer for at least five times repeatedly at an interval of 10 min. Washed membranes were further subjected to secondary antibody treatment with horseradish peroxidase (HRP)-conjugated for 2 h incubation at room temperature. The blots obtained were developed under an electrochemiluminescence (ECL) detection system (Bio-Rad Laboratory, Hercules, CA, USA). The expression of the proteins was analyzed using ImageJ 1.52a (U.S. National Institutes of Health, Bethesda, MD, USA). The relative density of the protein bands was normalized against the expression of β-actin, which was used as the control.

### 2.9. Statistical Analysis

The experimental results obtained in the study were analyzed statistically using GraphPad Prism software (version 5.0 GraphPad, Inc, San Diego, CA, USA). The results were expressed as the mean ± standard error of the mean (SEM) of triplicate samples. The significant differences between the groups were calculated using one way factorial analysis (ANOVA) followed by Bonferroni’s test. The *p*-value of <0.05 were considered statistically significant.

## 3. Results

### 3.1. Identification of Candidate Targets against Gastric Cancer

**Figure 2:**
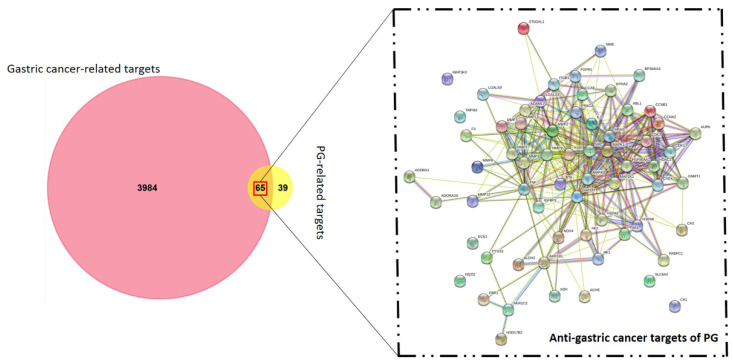
Venn diagram of gastric cancer and prunetin-5-O-glucoside targets. The candidate bio targets of prunetin-5-O-glucoside (PG) and gastric cancer were identified upon the Swiss target prediction and DisGeNET database. A protein-protein interaction (PPI) network of PG in gastric cancer targets was constructed for visualization of interactive targets.

The identification of anti-gastric cancer targets was performed after the search from the Swiss target prediction and DisGeNET databases. In total, 4049 gastric cancer hits and 104 putative PG targets were screened in the primary search. The co-relative targets were identified by construction of a Venn diagram using FunRich software. This resulted in a total of 65 common targets of PG against gastric cancer (Figure 2). The identified 65 targets was considered as the anti-gastric cancer targets of PG presented in Table S1 and was subjected to further analysis.

### 3.2. Functional Enrichment on the Identifiable Targets

**Figure 3:**
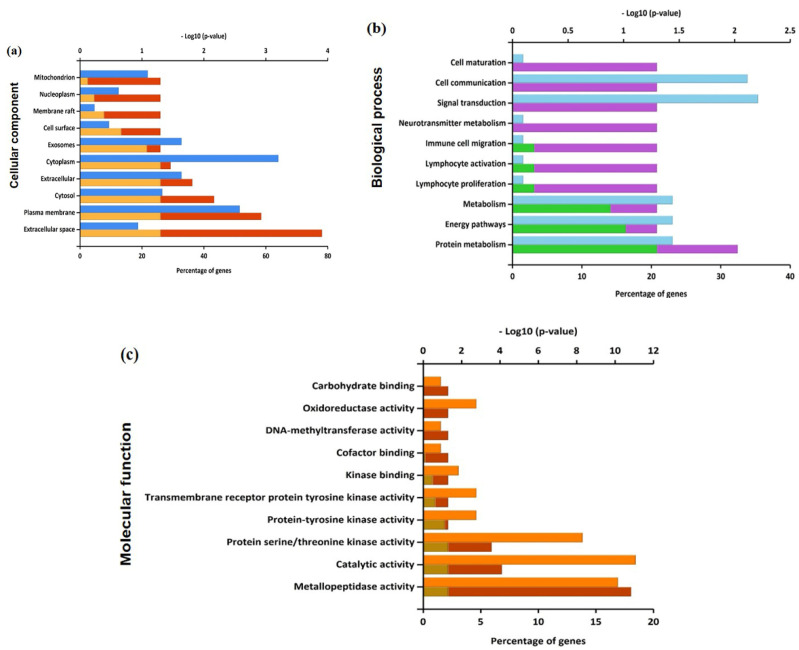
Enriched Gene Ontology in terms of (**a**) cellular component, (**b**) biological process, and (**c**) molecular function from predicted targets of PG against gastric cancer.

The biological functions and the signaling pathways enriched of all the 65 anti-gastric cancer targets of PG were analyzed using FunRich software. The Gene Ontology (GO) enrichment analysis was obtained based on three categories: biological function, cellular component, and molecular function. The top 10 significantly enriched GO terms among the 65 core targets were produced and depicted in Tables S2–S4. Figure 3a shows that a higher number of cellular component enrichment was identified in the extracellular space and cytoplasm. Figure 3b shows the enriched GO terms of biological process were associated with signal transduction and cell communication. Figure 3c shows the significant GO terms of molecular function were catalytic activity, protein serine/threonine kinase activity, and metallopeptidase activity, respectively.

**Figure 4:**
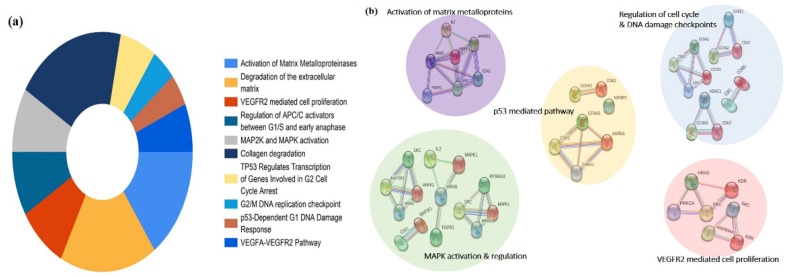
Pathway enrichment analysis of the predicted targets of PG. (**a**) Doughnut chart represents the top 10 enriched pathways of potential targets of PG identified by the Reactome pathway database. (**b**) Visual analysis of the protein-protein interaction network of the targets involved in the most significant enriched pathways.

The pathway enrichment analysis of the 65 potential targets were identified using the Reactome database. Upon screening, the top 10 significant pathways were found to be activation of matrix metalloproteinases, degradation of the extracellular matrix, VEGFR2 mediated cell proliferation, regulation of APC/C activators between G1/S and early anaphase, MAP2K and MAPK activation, collagen degradation, TP53 regulates transcription of genes involved in G2 cell cycle arrest, G2/M DNA replication checkpoint, p53-Dependent G1 DNA Damage Response, and VEGFA-VEGFR2 Pathway (Figure 4a). The targets among the pathway enrichment were grouped based on the interaction network constructed by the STRING database. The targets were grouped into the five most significant clusters that include the activation of matric metalloproteins, regulation of cell cycle and DNA damage checkpoints, p53 mediated pathway, MAPK activation and regulation, and VEGFR2 mediated cell proliferation, respectively (Figure 4b).

### 3.3. Network Interaction and Clustering Analysis of the Gastric Cancer Targets

**Figure 5:**
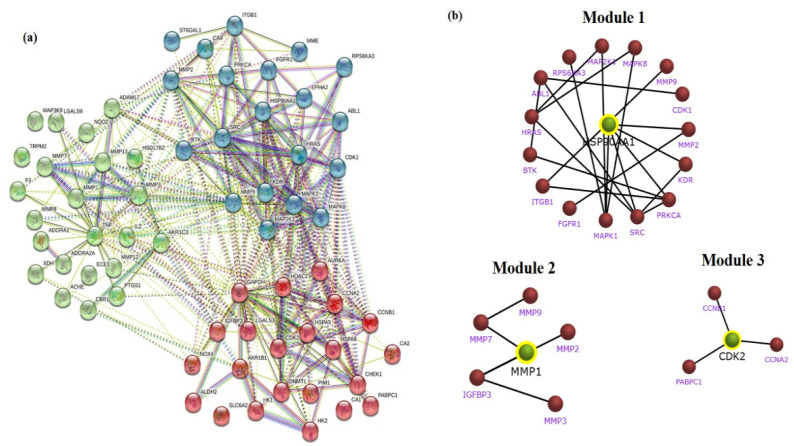
STRING clustering analysis and sub network module construction of crucial bio-targets. (**a**) Protein–protein interaction network of the anti-gastric cancer targets of PG. The targets are clustered based on the K-means clustering algorithm into three distinct groups of interactive networks. (**b**) Key subnetwork of the core targets constructed by module analysis.

The identifiable 65 targets of PG associated with GC was introduced into the STRING database to obtain the functional protein-association network. The protein–protein interaction (PPI) network of these anti-gastric cancer targets was constructed with 65 nodes and 357 edges obtained with a medium confidence score of 0.400 and enriched *p*-value of <1.0 × 10^−16^. The constructed network of interaction was subjected to clustering analysis using the K-means algorithm, which resulted in three distinct numbers of interactive networks as represented in Figure 5a. Furthermore, module construction of the three distinct networks was performed in FunRich software to identify the central interacting node of each network. Therefore, screening of each module was performed by analyzing the core target with the maximum degree of interaction among the majority of proteins. Correspondingly, the resultant showed three core target proteins among each module, namely, HSP90A1, CDK2, and MMP1, as shown in Figure 5b.

### 3.4. In Silico Validation of the Targets Using Molecular Docking

**Figure 6:**
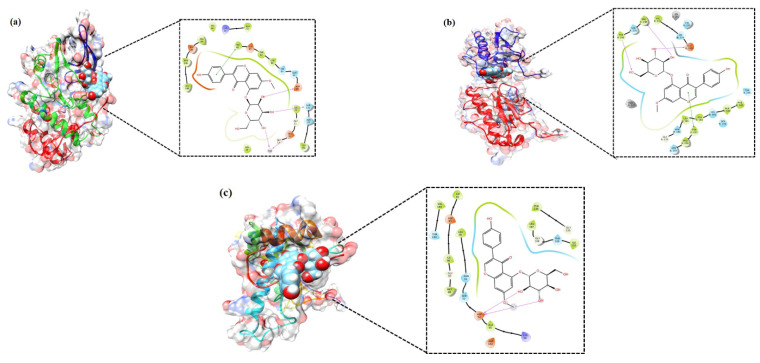
Molecular docking verification of the crucial targets of PG against gastric cancer. The three-dimensional structure of PG docked against the structure of the targets shown along with the 2D interaction diagram: (**a**) CDK2, (**b**) MMP1, and (**c**) HSP90.

The PG associated core targets were validated using molecular docking analysis. The three-dimensional structure of the MMP1 (PDB ID: 1CGL), CDK2 (PDB ID: 1AQ1), and HSP90 (PDB ID: 2VCJ) were downloaded from the RCSB Protein Data Bank. The target proteins were subjected to Glide docking based on the Standard Precision (SP) and Extra Precision (XP) scoring functions. The results showed that PG had good SP docking score with all three core targets ranging from −6.6. to −8.5 kcal/mol. The XP docking score of the best poses of PG with CDK2, MMP1 and HSP90 was −10.59 kcal/mol, −9.85 kcal/mol, and −9.43 kcal/mol, respectively. The ligand interaction diagram of PG with three targets are shown in Figure 6. The scoring values pertaining to SP, XP, and number of hydrogen bonds (HB), along with the interacting amino acid residues, are given in Table 1.

Analysis through molecular docking has become an important tool in the drug discovery process; however, it has certain limitations like poor scoring function and tackling the protein flexibility problem. For these reasons, the docking results should be improved by means of post-docking processing strategies [16]. Therefore, molecular docking was measured using the Prime/Molecular Mechanics-Generalized Born Surface Area (MM-GBSA) tool to calculate binding energy. Among the three targets, the free binding energy of PG with MMP1 was the lowest (−84.79 kcal/mol), followed by CDK2 (−76.97 kcal/mol) and HSP90 (−51.86 kcal/mol), which is shown in Table 2. The Coulomb energy, lipophilic energy, and van der Waals energy of all three complexes had negative values and showed favorable condition to the total free energy. However, solvation energy of all three complexes had positive values and contributed unfavorably toward total free energy.

### 3.5. Pharmacological Effect of PG by In Vitro Cell Culture Assessment and Morphological Examinations

**Figure 7:**
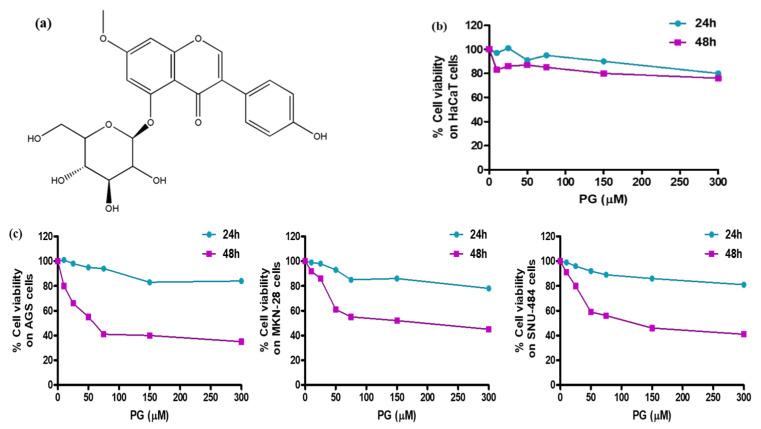
Cell viability assessment of PG treated gastric cancer cells and normal HaCaT cells. (**a**) Chemical structure of prunetinoside. (**b**) Cytotoxicity assessment by the MTT assay upon treatment with various concentrations (0, 10, 25, 50, 75, 150, and 300 μM) of PG at two different time intervals of 24 h and 48 h on normal human keratinocyte HaCaT cells. (**c**) Cytotoxicity assessment by the MTT assay upon treatment with various concentrations (0, 10, 25, 50, 75, 150, and 300 μM) of PG at two different time intervals of 24 h and 48 h on human gastric cancer cells AGS, MKN-28, and SNU-484 cells.

To further validate the bioinformatic findings, cell culture assessment was conducted to screen the cytotoxic effect of PG. Human gastric cancer cell lines—AGS, MKN-28, SNU-484 and normal human keratinocyte HaCaT cells—were used to evaluate the pharmacological activities of PG. The chemical structure of PG is shown in Figure 7a. HaCaT cells and gastric cancer cells treated with different concentrations of PG at two different time intervals of 24 h and 48 h were evaluated by the MTT assay. The results shown in Figure 7b indicate that at a maximum concentration of up to 300 µM of PG at both 24 h and 48 h does not affect the viability of HaCaT cells, whereas treatment with PG at the 48 h time interval showed a significant inhibition rate on the assessed gastric cancer cells (Figure 7c). These results indicate that the cytotoxic effect of PG is specific to cancer cells and does not affect the non-tumor cells.

The IC_50_ values of PG on the three gastric cancer cells at 24 h were found to be 82.2 µM in AGS cells, 76.5 µM in MKN-28, and 142.7 µM in SNU-484 cells. At 48 h, the IC_50_ values of PG were much stronger even at low concentrations, say 35.3 µM in AGS cells, 40.2 µM in MKN-28 cells, and 38.4 µM in SNU-484 cells, respectively (Table 3). In addition, it was observed that PG induced a stronger cytotoxic effect on AGS cells at comparably low concentration than the MKN-28 and SNU-484 cells. Based on this result, AGS cells more sensitive to PG were chosen for further in vitro experimentation.

Furthermore, the in silico pharmacokinetic properties of PG were predicted by its structure using SwissADME (Swiss Institute of Bioinformatics, Switzerland) and are provided in Table S6. The results showed that PG did not violate the Lipinski’s rule of 5, but showed a poor GI absorption property, which may be due to the presence of glucose moiety.

### 3.6. Induction of Cell Death by PG in AGS Cells through Western Blot and Morphological Examinations

**Figure 8:**
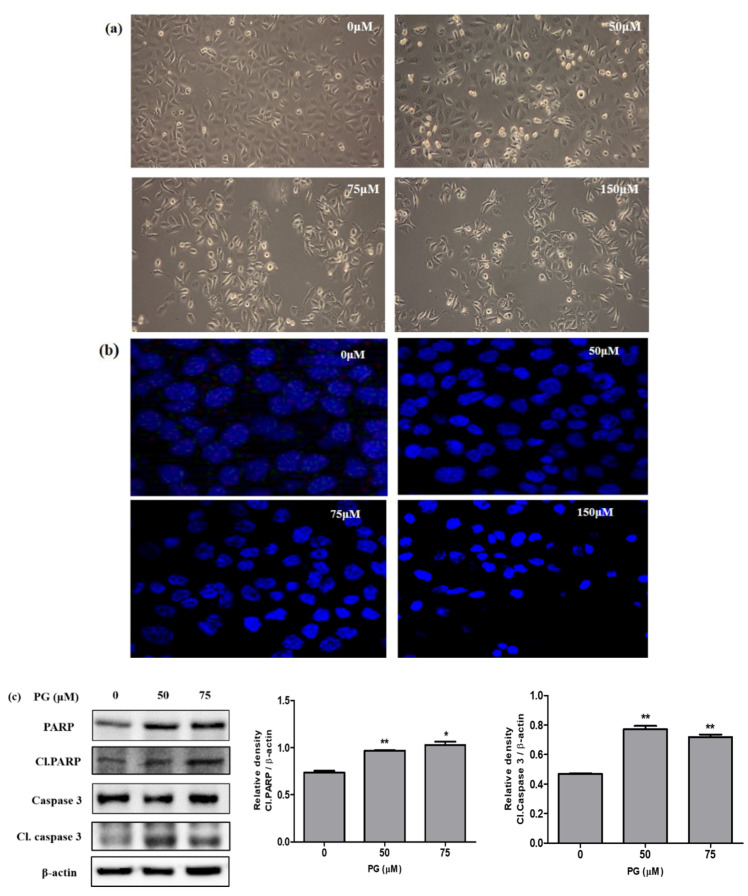
Cell death analysis by PG using morphological observations and western blot on PG treated AGS gastric cancer cells. (**a**) Morphological changes as observed under a light microscope showing cell death at indicated concentrations (0, 50, 75, and 150 μM) PG on AGS cells. (**b**) AGS cells treated with PG for 48 h and stained with DAPI followed by observation under confocal microscopy. (**c**) Protein expression of cell death hallmark proteins PARP, caspase 3, and its cleaved form on PRU treated AGS cells for 48 h. The expression levels of the target proteins were normalized against the control β-actin expression. The data are represented graphically based on its densitometry. Values are given as the mean ± standard error of the mean (SEM) of three independent experiments. * *p* < 0.05 vs. control and ** *p* < 0.01 vs. control.

Microscopical observations on PG treated AGS cells was performed using DAPI staining to investigate the cell death morphology. The results shown in Figure 8a represent the morphological changes on AGS cells treated with PG (50, 75, and 150 µM) for 48 h with an increase in floating cells, cell shrinkage, and cell ruptures, respectively. To further observe the cell nuclei damage in brief, PG treated AGS cells stained with DAPI were observed under confocal fluorescence microscopy. The results in Figure 8b show that there was an increased fluorescence, indicating fragmented nuclei on the PG treated group of cells representing the induction of cell death.

In addition, the induction of cell death caused by PG was studied by evaluating the protein expressions of important proteins PARP, cleaved PARP, caspase 3, and cleaved caspase 3. The event of apoptosis is coordinated by the activation of caspases that play a vital role as effectors or executioners in performing cell death. The activated form of caspase 3 tends to cleave several DNA-dependent protein kinases and poly ADP ribose polymerase (PARP) [24]. The cleaved form of the protein PARP is recognized as an important apoptotic marker that aids in bringing cellular damage through DNA cleavage and induces apoptotic cell death [25]. Results obtained from western blot revealed that the apoptotic marker proteins cleaved PARP and cleaved caspase 3 showed significant increased expression in AGS cells upon treatment with PG, as shown in Figure 8c. These data confirm that PG induces apoptotic cell death in AGS cells.

### 3.7. Evaluation of the Targets of by Its Protein and mRNA Expression Levels in Gastric Cancer

**Figure 9:**
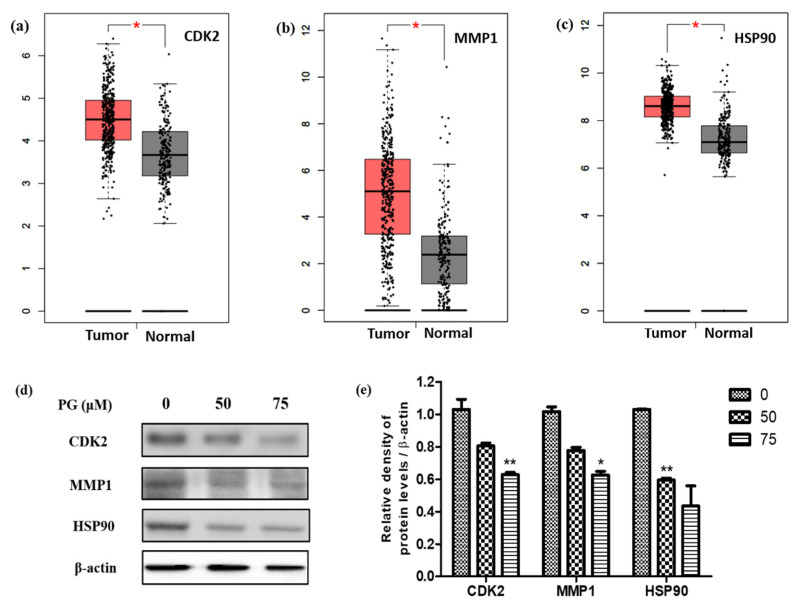
Validation of the crucial targets of PG against gastric cancer. Validation of the mRNA expression levels of (**a**) CDK2, (**b**) MMP1, and (**c**) HSP90 in STAD tissues and normal gastric tissues using GEPIA. These three box plots were based on 408 STAD samples (marked in red) and 211 normal samples (marked in gray). * *p* < 0.01 was considered statistically significant. STAD: stomach adenocarcinoma. (**d**) Protein expression of the targets CDK2, MMP1, and HSP90 on PRU treated AGS cells for 48 h. (**e**) The expression levels of the target proteins were normalized against the control β-actin expression. The data are represented graphically based on its densitometry. Values are given as the mean ± standard error of the mean (SEM) of three independent experiments. * *p* < 0.05 vs. control and ** *p* < 0.01 vs. control.

Based on the system pharmacology results, the mRNA expression levels of the obtained hub bio targets were explored using the GEPIA database. The analysis includes comparison of the three targets (CDK2, MMP1, and HSP90) between 408 stomach adenocarcinoma (STAD) samples and non-tumor stomach tissues. As demonstrated in Figure 9, the mRNA expression levels of CDK2 (Figure 9a), MMP1 (Figure 9b), and HSP90 (Figure 9c) were markedly upregulated in STAD tissue compared to the normal non-cancerous tissues. The results from the GEPIA database revealed that the mRNA expression levels of all three targets were significantly higher (*p* < 0.01) in STAD tissues than those in normal tissues.

After examining the mRNA expression levels, the three targets were validated by their protein expressions through western blot analysis on PG treated AGS cells. The results shown in Figure 9a show the decreased expression levels of the target proteins CDK2, MMP1, and HSP90 upon treatment with 50 µM and 75 µM concentrations of PG in AGS cells. Additionally, the relative expression graph of PG treatment and the target proteins was charted based on the results of the expression of the target proteins. The expression of the candidate target proteins was normalized with β-actin expression as the loading control and the appropriate significance level was calculated. As shown in Figure 9b, the relative expression of the target proteins showed a significant decrease in a dose-dependent manner compared to the control untreated group. These findings were consistent with the obtained mRNA data, which showed increased expression in gastric cancer tissues. Collectively, these results indicate that the identified hub targets are highly expressed in gastric cancer and are suppressed upon PG.

## 4. Discussion

Gastric cancer is the third leading cause of cancer cell death globally with a mortal rate in the advanced stages. The initial diagnosis, progression, and treatment of the disease condition is largely dismal [26]. Although chemotherapy and immunotherapy has very limited efficacy against the disease state, current emerging trends have turned toward the use of natural therapeutics in the developing research [27]. The advent of the big data era with the accumulation of evidence on the mechanisms of biological molecules as a progress of bioinformatic approaches provides a strong support in developing drugs through for network pharmacology [28]. The core concept of network pharmacology provides an extensive way to predict potential targets by analyzing their biological mechanisms from the perspective of network, which leads to the discovery of new active drugs from medicinal compounds [29].

In the current study, a bioinformatic investigation from web-based databases was utilized to explore the therapeutic mechanism of the flavonoid compound prunetin-5-O-glucoside (PG) as a treatment for gastric cancer through a network pharmacological approach. To the best of our knowledge, this is the first study integrating network pharmacology and molecular docking simulations to reveal the pharmacological mechanisms of PG against gastric cancer.

Primarily, identification of the therapeutic targets of PG in association with gastric cancer was performed through database screening. As a result, a total of 65 potential targets related to PG against gastric cancer were identified. The identified targets were studied for their functional enrichment through analysis by Gene Ontology (GO) terms. The majority of the enriched categories of targets were found to be related to signal transduction, cell communication process, and the molecular function of metallopeptidase activity. Furthermore, the pathway enrichment identified the top ten significantly enriched pathways. However, network clusters among the targets showed that major proteins were significantly executed in the regulation of cell cycle, MAPK activation and regulation, and activation of matrix metalloproteins.

Regulation of cell cycle is a frequently overactive pathway in cell growth that is associated with cell cycle proteins like cyclins and CHKs for regulating the uncontrolled proliferation in cancer [30]. Mitogen activated protein kinase (MAPK) pathways are a group of cascades that leads to activation of adequate signals in modulating cell growth. Due the upstream activating roles, MAPK plays key modulatory roles in the response of cancer cells [31]. The control of remodeling of the extracellular matrix (ECM) is merely important for cell invasion, growth, and metastatic growth of tumors. The activation of matrix metalloprotein (MMP) pathways is strongly associated with reconstruction of these ECM in terms of wound healing and repair. Thus, targeting MMP associated mechanisms by inhibitors are promising tool in treating cancer progression [32]. Therefore, it denotes that the anti-gastric cancer mechanism of PG may be benefited through regulating any of the above identified pathways.

The network construction on the targets and interaction among them revealed three interconnected subnetworks. Each cluster of subnetworks upon further analyses showed distinct modules with a key interacting network of hub genes. Among the three modules of hub interactions, the core targets were identified as HSP90, MMP1, and CDK2, respectively.

Matrix metalloproteinases family (MMPs) is an endopeptidase that degrades components present in the extracellular matrix. MMPs are also called as matrixins, which consists of 26 members ranging from MMP-1 to MMP-28 involved in the process such as wound healing, inflammatory response, and angiogenesis. Among these, MMP1 plays a major role in tumor invasion, neoangiogenesis, metastasis, and the proinflammatory process. Specifically, the extensive role of MMP1 in metastasis of gastric cancer has been reported [33]. Evidence on flavonoids like curcumin and its derivatives reports the suppression of migratory activity in cancer cells by inhibition of MMP1 [34]. Ginsenoside Rh1 (Rh1) inhibited colorectal cancer cell migration and invasion by partial inhibition of MMP1 and MMP3 [35]. Skullcapflavone II, a flavonoid derived from the root of Scutellaria baicalensis, has been reported to possess anticancer activity in human skin fibroblasts by suppressing MMP1 transcription [36].

Cyclin-dependent kinase (CDK) are one type of serine/threonine kinase protein systems involved in the cell cycle mechanism through activation at different phases of cell cycle events [37]. CDKs play a major role in the control of cell cycle progression during the complete cell division process. Deregulation of the cell cycle process is one of the major steps in the transformation of normal cells into cancerous cells. The CDK family protein has gained a crucial anticancer target for this reason [38]. Scutellarin is a natural flavone glycoside exerting anticancer activity by inhibiting the expression of proteins including cyclin D1, CDK2, Bcl2, MMP-2, and MMP-9 [39]. Shi et al. identified fluspirilene to be one of the candidate drugs in human cancer with an inhibitory mechanism of action against the CDK2 protein [40].

Heat-shock protein 90 (Hsp90) is a vital molecular chaperone ATPase-dependent protein that is involved in maturation, activation, and stabilization of various transcription factors [41]. The client proteins found in the Hsp90 family play fundamental roles in signal transduction, proliferation, cell cycle progression as well as in metastasis and tumor invasions [42]. Therefore, designing inhibitors for Hsp90 could induce the proteasomal degradation of Hsp90 client proteins, bringing about cell death in malignancies. Liu et al. reported that the derivative of quercetin (TL-2-8) induces cell death and immature mitophagy by inhibiting the function of AHA1/Hsp90 complex [43]. Higher levels of Hsp proteins (Hsp90 and Hsp70) elevated in breast cancer cells have been shown to be inhibited by the action of the flavonoid quercetin, causing apoptotic cell death [44]. Based on this literature, it can be inferred that all three core predicted targets play major roles in cancer progression and might be therapeutic targets for treating gastric cancer with PG.

Upon review of the literature, interestingly, it was found that the targets had a close association with the significantly enriched pathways: activation of matric metalloproteins, regulation of cell cycle and DNA damage checkpoints, p53 mediated pathway, MAPK activation and regulation, and VEGFR2 mediated cell proliferation. The target CDK2 is a vital protein kinase involved in cell cycle regulation induces arrest in G2 phase that has been effectively used as inhibitors to suppress cancer [45]. Similarly, CDK proteins are found to be activated in parallel with the p53 mediated signaling pathway, which tends to converge at the induction of apoptosis [46]. VEGF and VEGF receptor 2 (VEGFR-2) mediated cell proliferation pathways mediate major angiogenic function to maintain tissue homeostasis [47]. The target Hsp90, being a molecular chaperone protein, has been importantly involved in the maintenance of angiogenesis through these growth factor pathways. Thus, suppression of Hsp90 activity tends to inhibit the VEGFR-2 signals in cell proliferation [48]. The overexpression of the target MMP-1 is strongly associated with the activation of the MAPK pathway in a variety of cancer types [49]. A reported study has suggested that the overexpression of MMP-1 expression depends on the induction of MAPK through the activation of AP-1 transcriptional factors [50]. Thus, inhibition of MMP-1 through suppressing any of the MAPK pathway regulators—JNK/ERK—induces cell death in cancer cells. This indicates that PG could mediate these enriched pathway responses of the identified targets to bring cell death in gastric cancer.

Furthermore, cell culture assessment of PG treatment on human gastric cancer cells showed inhibition of cell growth and increased the cell death rate by dose-dependent regulations. Additionally, the non-cytotoxic effect of PG on normal HaCaT cells clearly denotes the cancer specific action of PG and is non-toxic to normal cells. Morphological evidence in the study showed visible cell death proportion through microscopic examinations. The upregulated protein expression on the cleavage of cell death marker proteins like PARP and caspase 3 on PG treated gastric cancer cell confirms the induction of apoptotic cell death.

As a validative perspective, the effect of PG on the core targets (HSP90, MMP1, and CDK2) were analyzed by in silico and in vitro approaches. The molecular interaction of the targets showed effective binding with PG in docking analysis. In addition, the evaluation of the hub targets analyzed for its mRNA expression through the GEPIA database showed high expressions in stomach cancer samples compared to the normal samples. In vitro validation of protein expression through western blot indicated significant downregulation of the targets in PG treated gastric cancer cells. In combination, these results indicate that the transcriptional expression levels of the predicted targets overexpressed in patients with GC and their translational expression prove its suppression upon treatment with PG.

## 5. Conclusions

In conclusion, the study identified the core targets and its biological functions, pathways, and the effect of PG on gastric cancer. The constructed network pharmacology revealed the significant interaction among the predicted targets that led to the identification of three core proteins (CDK2, MMP1, and HSP90) as the active bio targets of PG in gastric cancer. Molecular docking simulation showed the active binding potential between the targets and PG. Preliminary invitro studies prove the anticancer effect of PG and its potential to reduce the expression of the targets. Our study provides a theoretical foundation that PG might have specific therapeutic effects in gastric cancer and its identified pharmacological targets were experimentally verified, which might be potential hallmarks for treating gastric cancer.

## Figures and Tables

**Table 1:** Molecular docking studies of selected target protein complexed with prunetinoside and their binding energies.

Protein Target	SP (kcal/mol)	XP (kcal/mol)	HB (n) (kcal/mol)	Residues Involved in Hydrogen Bonds
CDK2	−7.21	−10.593	3	Asp-86, Gln-131, H_2_O
MMP1	−8.852	−9.848	3	Pro-238, Tyr-240, Gln-219
HSP90	−6.73	−9.429	2	Asp-54

HB (n)—number of hydrogen bonds.

**Table 2:** Post docking analysis using Prime/Molecular Mechanics-Generalized Born Surface (MM-GBSA) for docked complexes.

Protein-Ligand Complex	ΔG_Bind_	ΔG_Coul_	ΔG_Lipo_	ΔG_vdw_	ΔG_SolvGB_
CDK2	−76.97	−19.5621	−42.65	−47.80	36.53
MMP1	−84.79	−32.00	−39.49	−55.89	41.29
HSP90	−51.86	−1.89	−33.89	−55.14	37.89

All energies are expressed in kcal/mol. ΔG_Bind_—MM-GBSA free binding energy; ΔG_Coul_—Coulomb energy of the complex; ΔG_Lipo_—lipophilic energy of the complex; ΔG_vdw_—van der Waals energy of the complex; ΔG_SolvGB_—solvation energy of the complex.

**Table 3:** IC_50_ values of PG on three gastric cancer cell lines treated at two time intervals of 24 h and 48 h.

Gastric Cancer Cell Lines	AGS		MKN-28		SNU-484	
Time intervals	24 h	48 h	24 h	48 h	24 h	48 h
PG IC_50_ (μM)	82.2	35.3	76.5	40.2	142.7	38.4

All values are expressed in the micro molar scale (μM). The IC_50_ values were derived from smooth curve analysis in GraphPad Prism and were averaged from at least three independent experiments.

## Data Availability

The data presented in this study are available in this article and supplementary material.

## Supplementary Materials

The following are available online at https://www.mdpi.com/article/10.3390/cancers13081918/s1, Table S1: List of 65 potential anti-gastric cancer targets of PG, Table S2: Top 10 significantly enriched GO terms of cellular component associated with the identified anti-gastric cancer targets of PG, Table S3: Top 10 significantly enriched GO terms of molecular function associated with the identified anti-gastric cancer targets of PG, Table S4: Top 10 significantly enriched GO terms of biological process associated with the identified anti-gastric cancer targets of PG, Table S5: Top 10 significantly enriched pathways identified by the Reactome database and its associated anti-gastric cancer targets of PG, Table S6: Prediction of absorption, distribution, metabolism and excretion (ADME) profile of PG ADME profile of PG.
