# Supplementary material for: A Network Pharmacological Approach to Reveal the Pharmacological Targets and Its Associated Biological Mechanisms of Prunetin-5-O-Glucoside against Gastric Cancer
Source: Cancers (Basel). 2021 Apr 15;13(8):1918. doi: 10.3390/cancers13081918 (PMC8071515; doi:10.3390/cancers13081918)
Supplement: Supplementary file 1 [file cancers-13-01918-s001.zip › cancers-1183752-supplementary.pdf]

Supplementary Material: A Network Pharmacological Approach to Reveal the Pharmacological Targets and Its Associated Biological Mechanisms of Prunetin-5-O-Glucoside against Gastric Cancer

Preethi Vetrivel, Rajeswari Murugesan, Pritam Bhagwan Bhosale, Sang Eun Ha, Hun Hwan Kim, Jeong Doo Heo and Gon Sup Kim

Table S1. List of 65 potential anti-gastric cancer targets of PG.

Uniprot ID	Protein Name	Gene Name
P26358	DNA (cytosine-5)-methyltransferase 1	DNMT1
P09237	Matrilysin	MMP7
P28482	Mitogen-activated protein kinase 1	MAPK1
P01375	Tumor necrosis factor	TNF
P17936	Insulin-like growth factor-binding protein 3	IGFBP3
P17931	Galectin-3	LGALS3
P45983	Mitogen-activated protein kinase 8	MAPK8
P01112	GTPase HRas	HRAS
P11142	Heat shock cognate 71 kDa protein	HSPA8
P42330	Aldo-keto reductase family 1 member C3	AKR1C3
P00915	Carbonic anhydrase 1	CA1
P00918	Carbonic anhydrase 2	CA2
P05091	Aldehyde dehydrogenase, mitochondrial	ALDH2
Q13547	Histone deacetylase 1	HDAC1
P07900	Heat shock protein HSP 90-alpha	HSP90AA1
P35968	Vascular endothelial growth factor receptor 2	KDR
P03956	Interstitial collagenase	MMP1
P08253	72 kDa type IV collagenase	MMP2
P14780	Matrix metalloproteinase-9	MMP9
O14965	Aurora kinase A	AURKA
P11362	Fibroblast growth factor receptor 1	FGFR1
P11021	Endoplasmic reticulum chaperone BiP	HSPA5
P78536	Disintegrin and metalloproteinase domain-containing protein 17	ADAM17
P29317	Ephrin type-A receptor 2	EPHA2
P05556	Integrin beta-1	ITGB1
Q9NPC3	E3 ubiquitin-protein ligase CCNB1IP1	CCNB1
P24941	Cyclin-dependent kinase 2	CDK2
P14784	Interleukin-2 receptor subunit beta	IL2
Q9NPH5	NADPH oxidase 4	NOX4
P04406	Glyceraldehyde-3-phosphate dehydrogenase	GAPDH
P45452	Collagenase 3	MMP13
Q02750	Dual specificity mitogen-activated protein kinase kinase 1	MAP2K1
O14757	Serine/threonine-protein kinase Chk1	CHEK1
P00519	Tyrosine-protein kinase ABL1	ABL1

O00182	Galectin-9	LGALS9
P11309	Serine/threonine-protein kinase pim-1	PIM1
Q16790	Carbonic anhydrase 9	CA9
P13726	Tissue factor	F3
P52789	Hexokinase-2	HK2
P08473	Neprilysin	MME
P08254	Stromelysin-1	MMP3
P39900	Macrophage metalloelastase	MMP12
P23219	Prostaglandin G/H synthase 1	PTGS1
P15907	Beta-galactoside alpha-2,6-sialyltransferase 1	ST6GAL1
P12931	Proto-oncogene tyrosine-protein kinase Src	SRC
P47989	Xanthine dehydrogenase/oxidase	XDH
P16152	Carbonyl reductase [NADPH] 1	CBR1
P20248	Cyclin-A2	CCNA2
P06493	Cyclin-dependent kinase 1	CDK1
P30542	Adenosine receptor A1	ADORA1
P29274	Adenosine receptor A2a	ADORA2A
P42892	Endothelin-converting enzyme 1	ECE1
P15121	Aldo-keto reductase family 1 member B1	AKR1B1
P19367	Hexokinase-1	HK1
P37059	Estradiol 17-beta-dehydrogenase 2	HSD17B2
P22303	Acetylcholinesterase	ACHE
P22894	Neutrophil collagenase	MMP8
P16083	Ribosyldihydronicotinamide dehydrogenase [quinone]	NQO2
P17252	Protein kinase C alpha type	PRKCA
Q06187	Tyrosine-protein kinase BTK	BTK
O94759	Transient receptor potential cation channel subfamily M member 2	TRPM2
P80192	Mitogen-activated protein kinase kinase kinase 9	MAP3K9
P51812	Ribosomal protein S6 kinase alpha-3	RPS6KA3
P23975	Sodium-dependent noradrenaline transporter	SLC6A2
P11940	Polyadenylate-binding protein 1	PABPC1

Table S2. Top 10 significantly enriched GO terms of cellular component associated with the identified anti-gastric cancer targets of PG.

Cellular Component	No. of Genes in the Dataset	Percentage of Genes	Fold Enrichment	*p*-value (Hypergeometric Test)
Extracellular space	12	18.75	6.75858676	1.58584×10^{-7}
Plasma membrane	33	51.5625	2.15722204	1.51231×10^{-6}
Cytosol	17	26.5625	3.28292028	8.71431×10^{-6}
Extracellular	21	32.8125	2.61737412	1.96317×10^{-5}
Cytoplasm	41	64.0625	1.64036289	4.40369×10^{-5}
Exosomes	21	32.8125	2.33808643	0.000106361
Cell surface	6	9.375	6.66506558	0.000275865
Membrane raft	3	4.6875	19.0041467	0.000522182
Nucleoplasm	8	12.5	4.05584644	0.000750549
Mitochondrion	14	21.875	2.52996176	0.000954103

Table S3. Top 10 significantly enriched GO terms of molecular function associated with the identified anti-gastric cancer targets of PG.

Molecular Function	No. of Genes in the Dataset	Percentage of Genes	Fold Enrichment	*p*-value (Hypergeometric Test)
Metallopeptidase activity	11	16.9230769	30.3893238	6.41797×10^{-14}
Catalytic activity	12	18.4615384	6.29392119	3.52986×10^{-7}
Protein serine/threonine kinase activity	9	13.8461538	8.34530005	1.20605×10^{-6}
Protein-tyrosine kinase activity	3	4.61538461	22.0783551	0.000339501
Transmembrane receptor protein tyrosine kinase activity	3	4.61538461	14.983008	0.001065435
Kinase binding	2	3.07692307	35.0028022	0.001471155
Cofactor binding	1	1.53846153	278.803415	0.003586207
DNA-methyltransferase activity	1	1.53846153	70.2223065	0.014269021
Oxidoreductase activity	3	4.61538461	5.21208794	0.020110712
Carbohydrate binding	1	1.53846153	40.1699642	0.024839046

Table S4. Top 10 significantly enriched GO terms of biological process associated with the identified anti-gastric cancer targets of PG.

Biological Process	No. of Genes in the Dataset	Percentage of Genes	Fold Enrichment	*p*-value (Hypergeometric Test)
Protein metabolism	15	23.07692308	3.16294538	5.27536×10^{-5}
Energy pathways	15	23.07692308	2.5625124	0.000537893
Metabolism	15	23.07692308	2.48638354	0.000737945
Lymphocyte proliferation	1	1.538461538	278.788033	0.003586405
Lymphocyte activation	1	1.538461538	278.788033	0.003586405
Immune cell migration	1	1.538461538	278.788033	0.003586405
Neurotransmitter metabolism	1	1.538461538	140.087519	0.007160144
Signal transduction	23	35.38461538	1.63062947	0.007903902
Cell communication	22	33.84615385	1.65260115	0.00825614
Cell maturation	1	1.538461538	70.2184322	0.014269804

Table S5. Top 10 significantly enriched pathways identified by Reactome database and its associated anti-gastric cancer targets of PG.

S. No	Pathway Description	Number of Targets
1	Activation of matrix metalloproteinases	MMP7; MMP1; MMP2; MMP9; MMP13; MMP3;MMP8;
2	Degradation of the extracellular matrix	MMP7; MMP1; MMP2; MMP9; MMP13; MMP3; MMP12; MMP8;
3	VEGFR2 mediated cell proliferation	HRAS; KDR; SRC; PRKCA;
4	Regulation of APC/C activators between G1/S and early anaphase	CCNB1; CDK2; CCNA2; CDK1;
5	MAP2K and MAPK activation	MAPK1; HRAS; MAP2K1; SRC;
6	Collagen degradation	MMP7; MMP1; MMP2; MMP9; ADAM17; MMP13; MMP3; MMP12; MMP8;
7	TP53 regulates transcription of genes involved in G2 cell cycle arrest	AURKA; CCNB1; CDK1;
8	G2/M DNA replication checkpoint	CCNB1; CDK1; CHEK1
9	p53-Dependent G1 DNA Damage Response	CDK2; CCNA2;
10	VEGFA-VEGFR2 Pathway	HSP90AA1; KDR; SRC;

Table S6. Prediction of Absorption, Distribution, Metabolism and Excretion (ADME) profile of PG.

ADME Parameters	Predicted Value	ADME Parameters	Predicted Value	ADME Parameters	Predicted Value
MW	446.4	ESOL Class	Soluble	CYP2C9 inhibitor	No
#Rotatable bonds	5	Ali Log S	−3.56	CYP2D6 inhibitor	No
#H-bond acceptors	10	Ali Solubility (mg/mL)	1.24×10^{-01}	CYP3A4 inhibitor	Yes
#H-bond donors	5	Ali Solubility (mol/L)	2.78×10^{-04}	log Kp (cm/s)	−8.57
MR	110.58	Ali Class	Soluble	Lipinski #violations	0
TPSA	159.05	Silicos-IT LogSw	−3.38	Ghose #violations	0
iLOGP	1.95	Silicos-IT Solubility (mg/mL)	1.85×10^{-01}	Veber #violations	1
XLOGP3	0.64	Silicos-IT Solubility (mol/L)	4.15×10^{-04}	Egan #violations	1
WLOGP	0.35	Silicos-IT class	Soluble	Muegge #violations	1
MLOGP	−1.39	GI absorption	Low	Bioavailability Score	0.55
Silicos-IT Log P	0.89	BBB permeant	No	PAINS #alerts	0
Consensus Log P	0.49	Pgp substrate	No	Brenk #alerts	0
ESOL Log S	−3.05	CYP1A2 inhibitor	No	Leadlikeness #violations	1
ESOL Solubility (mg/mL)	3.97×10^{-01}	CYP2C19 inhibitor	No	Synthetic Accessibility	5.29
